# Myelin Basic Protein and Cardiac Sympathetic Neurodegeneration in Nonhuman Primates

**DOI:** 10.1155/2021/4776610

**Published:** 2021-10-04

**Authors:** Jeanette M. Metzger, Helen N. Matsoff, Don Vu, Alexandra D. Zinnen, Kathryn M. Jones, Viktoriya Bondarenko, Heather A. Simmons, Colleen F. Moore, Marina E. Emborg

**Affiliations:** ^1^Wisconsin National Primate Research Center, University of Wisconsin-Madison, Madison, WI 53715, USA; ^2^Occupational Therapy Program, University of Wisconsin-Madison, Madison, WI 53715, USA; ^3^Department of Psychology, University of Wisconsin-Madison, Madison, WI 53715, USA; ^4^Department of Medical Physics, University of Wisconsin-Madison, Madison, WI 53715, USA

## Abstract

Minimal myelination is proposed to be a contributing factor to the preferential nigral neuronal loss in Parkinson's disease (PD). Similar to nigral dopaminergic neurons, sympathetic neurons innervating the heart have long, thin axons which are unmyelinated or minimally myelinated. Interestingly, cardiac sympathetic loss in PD is heterogeneous across the heart, yet the spatial relationship between myelination and neurodegeneration is unknown. Here, we report the mapping of myelin basic protein (MBP) expression across the left ventricle of normal rhesus macaques (*n* = 5) and animals intoxicated with systemic 6-OHDA (50 mg/kg iv) to model parkinsonian cardiac neurodegeneration (*n* = 10). A subset of 6-OHDA-treated rhesus received daily dosing of pioglitazone (5 mg/kg po; *n* = 5), a PPAR*γ* agonist with neuroprotective properties. In normal animals, MBP-immunoreactivity (-ir) was identified surrounding approximately 14% of axonal fibers within nerve bundles of the left ventricle, with more myelinated nerve fibers at the base level of the left ventricle than the apex (*p* < 0.014). Greater MBP-ir at the base was related to a greater number of nerve bundles at that level relative to the apex (*p* < 0.05), as the percent of myelinated nerve fibers in bundles was not significantly different between levels of the heart. Cardiac sympathetic loss following 6-OHDA was associated with decreased MBP-ir in cardiac nerve bundles, with the percent decrease of MBP-ir greater in the apex (84.5%) than the base (52.0%). Interestingly, cardiac regions and levels with more MBP-ir in normal animals showed attenuated sympathetic loss relative to areas with less MBP-ir in 6-OHDA + placebo (*r* = −0.7, *p* < 0.014), but not in 6-OHDA + pioglitazone (*r* = −0.1) subjects. Our results demonstrate that myelination is present around a minority of left ventricle nerve bundle fibers, is heterogeneously distributed in the heart of rhesus macaques, and has a complex relationship with cardiac sympathetic neurodegeneration and neuroprotection.

## 1. Introduction

Cardiac sympathetic neurodegeneration is a common feature of Parkinson's disease (PD) that may precede the hallmark nigral dopaminergic neuron loss and onset of motor symptoms characteristic of the disease [1]. It is a key component of cardiac dysautonomia and affects approximately 80% of PD patients. Orthostatic hypotension, the inability to regulate blood pressure with changes in body position, is a typical symptom of PD cardiac dysautonomia that causes dizziness and syncope, which in turn increases the risk of falls and injury [1, 2]. The loss of sympathetic fibers in the heart is nonuniform and progresses over time [3]. Cardiac sympathetic denervation typically starts in the apical level and the inferior wall of the left ventricle [4, 5] and may ultimately become extensive and uniform [6]. The mechanisms driving the anatomically heterogenous and progressive cardiac sympathetic loss in PD are unknown.

Minimal myelination of substantia nigra neurons is proposed to be a contributing factor to the preferential nigral cell loss in PD. Dopaminergic neurons of the substantia nigra typically project into the striatum and have long, thin axons that are unmyelinated or have only a thin myelin sheath [7, 8]. Cardiac sympathetic axons emerge from the paravertebral sympathetic ganglia and travel as cardiac nerves before joining with parasympathetic fibers to form the cardiac plexus at the base of the heart [9]. From the cardiac plexus, axons distribute throughout the heart in nerve bundles before reaching myocardial targets as single fibers. Similar to the axons of dopaminergic nigral neurons, these postganglionic sympathetic axons are also typically described as poorly or unmyelinated. Interestingly, a report analyzing myelination of sympathetic fibers in the anterior wall of the left ventricle found greater loss of unmyelinated compared to myelinated fibers in PD [10], suggesting that myelination may also have a role in cardiac sympathetic neurodegeneration in PD.

Cardiac sympathetic neurodegeneration can be modeled in rhesus monkeys by systemic administration of 6-hydroxydopamine (6-OHDA) [11, 12]. The toxin enters neurons through catecholamine reuptake transporters at the axon terminals and induces cell death by increasing oxidative stress through autooxidation and mitochondrial complex I inhibition, which triggers recruitment of immune cells [13]. Like PD patients, 6-OHDA-treated monkeys present heterogenous loss of cardiac sympathetic innervation, with the most loss in the apex and inferior wall of the left ventricle [11, 12, 14, 15]. Administration of the peroxisome proliferator receptor gamma (PPAR*γ*) agonist pioglitazone to 6-OHDA treated monkeys induces mild neuroprotection limited to the anterior-lateral region of the base and the septal region of the apex, supporting the concept of anatomical differences in susceptibility to neurodegeneration and neuroprotection [14]. However, the relationship between cardiac regional vulnerability to sympathetic loss and the distribution of myelinated fibers throughout the heart has yet to be described.

Myelin is an extended and modified glial cell plasma membrane with both protein and lipid components that wraps around the axons of certain populations of neurons, resulting in improved electrical conduction efficiency and trophic support [16, 17]. Myelin basic protein (MBP), the main protein component of myelin, is critical for the development and maintenance of axonal myelination and serves as a marker of the presence of myelin in tissue. MBP is a structural protein that binds the negatively charged surfaces of the myelin bilayer to create layered membrane stacks [18, 19]. MBP and myelin are produced in both the central and peripheral nervous systems, by oligodendrocytes and Schwann cells, respectively.

The overall aim of this report was to assess the role of myelin in cardiac sympathetic neurodegeneration. We first characterized and mapped MBP expression in the cardiac left ventricle of normal rhesus macaques and then evaluated myelination in 6-OHDA-treated monkeys dosed with placebo or pioglitazone. Finally, we compared the cardioanatomical distribution of myelin and sympathetic innervation between treatment groups to assess whether there is a relationship between the pattern of MBP expression and sympathetic neurodegeneration and preservation in the heart.

## 2. Materials and Methods

### 2.1. Compliance with Ethical Standards

The present study was performed in strict accordance with the recommendations in the National Research Council Guide for the Care and Use of Laboratory Animals (8^th^ edition, 2011) in an AAALAC accredited facility (Wisconsin National Primate Research Center (WNPRC), University of Wisconsin-Madison). Experimental procedures were approved by the Institutional Animal Care and Use Committee (IACUC) at the University of Wisconsin-Madison (experimental protocol G00705). The naïve (normal control) monkey tissues were obtained from the WNPRC tissue bank (original experimental protocols wprc00 and G005273). All efforts were made to minimize the number of animals used and to ameliorate any distress.

### 2.2. Subjects

Cardiac tissues from 15 adult, male rhesus monkeys (*Macaca mulatta*) were used in this project. The animals were individually housed in Group 3 or Group 4 enclosures (cage floor area 4.3 ft^2^ or 6.0 ft^2^ per animal, height 30 or 32 in.) in accordance with the Animal Welfare Act and its regulations and the Guide for the Care and Use of Laboratory Animals (8th edition, 2011) with a 12-hour light/dark cycle. Throughout the study, the animals were monitored twice daily by an animal research or veterinary technician for evidence of disease or injury (e.g., inappetence, dehydration, diarrhea, lethargy, trauma, etc.), and body weight was monitored to ensure animals remained in properly sized cages. Animals were fed commercial nonhuman primate chow (2050 Teklad Global 20% Protein Primate Diet, Harlan Laboratories, Madison, WI) twice daily, supplemented with fruits or vegetables and a variety of forage items and received ad libitum water. Nonhuman primate chow soaked in a protein-enriched drink (Ensure©, Abbott Laboratories, Abbott Park, IL) was offered to stimulate appetite as needed. Environmental enrichment opportunities were presented a minimum of 5 days per week. Enrichment toys and puzzles were provided on a rotational schedule to ensure novel stimulation and to engage animals' species-typical curiosity and manipulatory behavior.

The animals were part of previously published studies [14, 15]. To model cardiac sympathetic neurodegeneration, monkeys received systemic 6-OHDA (50 mg/kg iv) [11, 14]. Twenty-four hours later, the animals were randomly and blindly assigned to receive daily oral dosing of placebo (*n* = 5; 6.2–13 years old; 9.8–12.3 kg) or pioglitazone (5 mg/kg; *n* = 5; 5.6–11.4 years old; 9.4–10.6 kg). The normal control rhesus monkey tissues (*n* = 5; 6.8–12.4 years old; 9.6–12.6 kg) were obtained from the WNPRC tissue bank. Donors were selected by matching them to the baseline condition of the 6-OHDA-treated monkeys. The number of subjects per group was defined by our previous cardiac studies [11, 12, 14, 15] in which *n* = 5 found statistically significant loss of cardiac innervation. Only male monkeys were used due to the higher impact of PD in the male population (https://www.cdc.gov/mmwr/volumes/68/wr/mm6835a6.htm).

### 2.3. Necropsy and Tissue Preparation

All monkeys (6-OHDA-treated and normal controls) were euthanized by transaortic perfusion and tissues collected following previously published methods [14, 15]. In brief, animals were anesthetized with ketamine hydrochloride (10 mg/kg, im) followed by pentobarbital sodium (minimum of 25 mg/kg, iv) and perfused through the left atrium with heparinized phosphate-buffered saline (PBS), followed by 4% paraformaldehyde (PFA). Hearts were postfixated in 4% PFA for 24 hours followed by 70% ethanol. The hearts were cut into 4 mm sections in a transverse plane creating 8 levels from the apex to the base using a calibrated polymethyl methacrylate slice apparatus [14]. After a 1 mm punch was placed in the anteroseptal myocardium to mark orientation, the sections were blocked in paraffin. The blocks were cut on a standard rotary microtome in 5 *μ*m section thickness and mounted on positively charged slides.

### 2.4. General Immunohistochemistry

Cardiac immunohistochemistry against myelin basic protein (MBP; 1 : 200; Abcam AB7349), protein gene product 9.5 (PGP9.5; 1 : 200; Abcam 53057), and tyrosine hydroxylase (TH; 1 : 200; Immunostar 22941) was performed as previously described [14, 15]. In brief, cardiac sections were deparaffinized and treated for heat antigen retrieval in a microwave for 6 minutes at 100% power followed by 6 minutes at 80% power and left to cool for 1 hour at room temperature. The sections were then washed, and endogenous peroxidase activity was blocked by incubation of 30% H_2_O_2_ and methanol. Nonspecific binding sites were blocked with Super Block (ScyTek, Logan, UT) for 30 minutes at room temperature and incubated overnight with a primary antibody diluted in blocking buffer plus 0.1% Triton-X. The sections were then incubated in species-appropriate biotinylated secondary antibody (1 : 200; Vector Lab BA-2000, BA-1000, or BA-9400), followed by avidin-biotin-complex peroxidase (VECTASTAIN Elite ABC HRP Kit, Vector Laboratories, Burlingame, CA), and visualized with a commercial 3,3′-diaminobenzidine (DAB) kit (Vector Laboratories, Burlingame, CA). TH and PGP9.5 immunolabeled cardiac sections were counterstained with Hematoxylin (VWR, Radnor, PA); all slides were dehydrated, and coverslipped (Cytoseal mounting media, Thermo Scientific, Waltham, MA). Negative controls for cardiac immunostainings were performed by omitting the primary antibodies. Tissues from all three treatment groups were processed in parallel for each staining to minimize bias.

### 2.5. Immunofluorescence

Immunofluorescence stainings were performed to identify myelinated nerve fibers (MBP-immunoreactivity (-ir) surrounding PGP9.5-ir) or myelinated sympathetic nerve fibers (MBP-ir surrounding PGP9.5-ir and TH-ir). Cardiac slides were deparaffinized, and antigen retrieval was performed as described above. Tissue was blocked with Super Block (ScyTek, Logan, UT) and incubated overnight at room temperature in primary antibodies against MBP (1 : 100; Abcam AB7349) and protein gene product 9.5 (PGP9.5; 1 : 200, Abcam 53057) or antibodies against MBP, PGP9.5, and TH (1 : 200; Immunostar 22941) diluted in blocking buffer plus 0.1% Triton-X. The sections were then incubated with species-appropriate Alexa Fluor-conjugated secondary antibodies (1 : 1000; Invitrogen A21207, Invitrogen A21208, or Abcam AB15107) and coverslipped with mounting media with DAPI (Vector Laboratories, Burlingame, CA).

### 2.6. Quantification of MBP, PGP9.5, and TH Expression in Myocardial Nerve Bundles

MBP-ir, PGP9.5-ir, and TH-ir in myocardial nerve bundles across the left ventricle were blindly evaluated at three levels (base, middle, apex), subdivided into four regions (septal, anterior, lateral, inferior) (Supplementary Figure S1). Protein expression was quantified within cardiac nerve bundles identified in the myocardium. A myocardial nerve bundle was defined as a collection of fibers >30 *μ*m^2^. Bundles present in the pericardium/epicardium were excluded from the analysis. All brightfield microscopy and image collection was performed using a Zeiss Axio Imager M2 microscope with images captured with a 63x objective.

**Figure 1:**
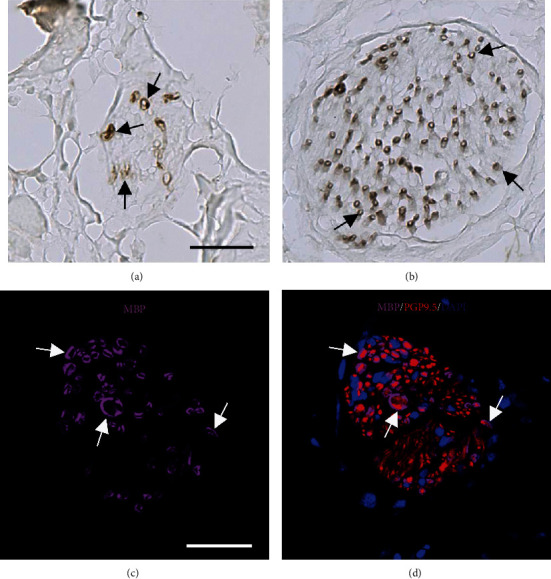
The myelin marker MBP surrounds axons in myocardial nerve bundles in the heart of rhesus macaques. Brightfield ((a, b); brown color) and fluorescent ((c, d); pink color) immunolabeling of MBP (arrows). MBP-immunoreactivity (-ir) surrounds a minority of PGP9.5-ir neuronal axons (red). Scale bars = 50 *μ*m. MBP, myelin basic protein; PGP9.5, protein gene product 9.5.

MBP-ir was analyzed by searching across each MBP immunostained cardiac section at 10x for the presence of bundles with MBP-ir. Each bundle that appeared positive was then examined at 63x for further quantification. MBP-ir in nerve bundles was defined as the presence of dark brown “spots” (MBP-ir spots) (Figures 1(a) and 1(b)). Data collected were the number of bundles with MBP-ir spots in each cardiac region, the number of MBP-ir spots per bundle, and the total number of MBP-ir spots overall, per region and level for each animal. The reliability of the MBP-ir spot counting method was assessed as intrarater reliability, in which one investigator performed three ratings on a subset of regions and levels in select animals; both the second (Spearman *r* = 0.69; *p* < 0.001) and third (Spearman *r* = 0.78; *p* < 0.001) round of ratings showed acceptable reliability and significantly correlated with the first rating (Supplementary Figure S2).

PGP9.5-ir was evaluated to generate data on the number of bundles in each region of each level and data on the size (area) of nerve bundles (Supplementary Figure S3). PGP9.5-ir bundles were counted when >30 *μ*m^2^. To evaluate the size of nerve bundles, images of 6 bundles per region per level were captured at 63x, and ImageJ was used to draw an outline around the perimeter of the bundle. High-resolution scanned images of the PGP9.5 immunostained slides were also used to calculate the size (area) of the cardiac tissue included in each region (Supplementary Figure S1) in ImageJ.

Analysis of TH expression in cardiac nerve bundles in each level and region was previously reported [14, 15]. In brief, cardiac nerve bundle images were captured as above and TH-ir was quantified as percent of the bundle area with immunostaining (percent area above threshold, %AAT).

### 2.7. Quantification of Percentage of Myelinated Fibers in Nerve Bundles

The percentage of myelinated fibers in cardiac nerve bundles was estimated in the anterior region of the base and apex levels of the left ventricle by analyzing PGP9.5-ir axons and MBP-ir spots in three normal subjects. Cardiac nerve bundle images from MBP and PGP9.5 double-labeled immunofluorescence slides were captured at 20x using a Nikon A1R-HD confocal microscope. As individual PGP9.5-ir fibers were difficult to identify at this magnification, the average MBP-ir spot size was assumed to be approximately equal to the average size of a nerve fiber. The average MBP-ir spot size was calculated by outlining 38 individual MBP-ir spots in randomly selected nerve bundles in a representative normal subject at the base and apex of the heart using the Free-hand Tool and the Measure function in NIH ImageJ software. The approximate number of fibers in each bundle was calculated by dividing the bundle area by the average area of a fiber. The number of MBP-ir spots was compared to the estimated number of fibers to obtain a percentage of myelinated fibers for each bundle in these three subjects.

### 2.8. Statistical Analysis

Data collection and analysis were performed by investigators blind to the treatment groups. A *p* value of <0.05 was accepted as significant. Averages are given as either “mean ± SEM” where SEM is the standard error of the mean or as “median, IQR” where IQR is the interquartile range, as appropriate for the data distribution.

MBP-ir spot data was analyzed to determine the distribution of myelinated fibers across cardiac anatomy and to compare between treatment groups. Because these data are counts, they are nonnormally distributed with overdispersion (i.e., the variance is larger than the mean) and zero inflation (many zeros). Therefore, generalized estimation equations (GEE) models were used with the Poisson family and exchangeable correlation structure in the R package “geepack” following examples of analysis of similar datasets [20, 21]. Statistically significant effects of treatment group, cardiac level, or cardiac region were further broken down by pairwise comparisons using Friedman analysis of variance for ranks in BMDP [22]. Using the Friedman tests, the cardiac levels were compared at each region separately and the cardiac regions were compared at each level separately in each group, and the *p* values were adjusted by the Bonferroni method.

All other statistical analyses were performed in GraphPad Prism (version 8.0, GraphPad Software). Interrater reliability was evaluated using the nonparametric Spearman's rank-order correlation and reported as the Spearman “r” and two-tailed *p* value. Data on the percentage of myelinated fibers in nerve bundles was compared between the base and apex layers using the Student's *t*-test. This same data set was evaluated by Spearman's rank-order correlation to test for a relationship between the size (area) of these bundles and the number of MBP-ir spots or percentage of fibers myelinated. The size (area) of PGP9.5-ir nerve bundles in control animals was compared using ANOVA as 3 (level: base, middle, apex) × 4 (region: septal, anterior, lateral, inferior) with repeated measures on level and region, the Geisser-Greenhouse correction for lack of sphericity, and post hoc analysis using Bonferroni multiple comparisons. Similar statistical analysis was performed to compare the number of bundles and percentage of bundles with any MBP-ir spots across cardiac anatomy. The size (area) of the cardiac regions was analyzed by fitting a mixed-effects model (REML) rather than ANOVA due to technical difficulties preventing data from one cardiac level in one animal from being included; multiple comparisons between cardiac regions and levels were corrected via Bonferroni's multiple comparisons test. Correlations between MBP-ir spot count or percent loss of MBP-ir spots and loss of sympathetic markers were performed using Spearman's rank-order correlation.

## 3. Results

### 3.1. In Normal Rhesus Macaques, Myelinated Axons Were Heterogeneously Distributed across the Cardiac Left Ventricle

The myelin marker MBP was identified in myocardial nerve bundles throughout the left ventricle. MBP-ir could be seen as dark brown “spots” with a regular circle or oval shape, suggesting myelin surrounding axons (Figures 1(a) and 1(b)). Immunofluorescent labeling of both MBP and the pan-neuronal marker PGP9.5 confirmed the presence of axons centered in the spots of myelin (Figures 1(c) and 1(d)). Within nerve bundles, MBP-ir spot distribution was diverse, with myelinated axons found near the perimeter or center of bundles, as individual spots or clusters, with no set pattern.

**Figure 2:**
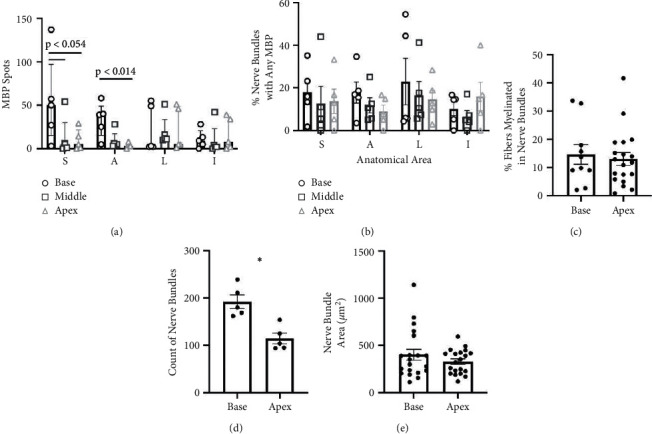
Cardiac MBP-immunoreactive (-ir) spot number in normal rhesus macaques follows a base to apex gradient. Bar graph (a) of the total number of MBP-ir spots (median ± interquartile range) counted in each anatomical area (3 levels ∗ 4 regions) in the normal, control animals. Bar graph (b) of the estimated percentage of bundles with any MBP-ir spots (mean ± standard error of the mean) in each anatomical area in control animals. Bar graphs of the average (mean ± standard error of the mean) percentage of fibers myelinated in bundles (c), the number of nerve bundles (d), or size of nerve bundles (e) in the base and apex levels of control animals; each point represents one myocardial nerve bundle. ^*∗*^, *p* < 0.001 septal region, *p* < 0.05 inferior region; see Supplementary Figure S4 for more details. Note that (d) and (e) include data from all regions in the base and apex levels, while (c) includes only the anterior region. MBP, myelin basic protein; S, septal; A, anterior; L, lateral; I, inferior.

Myelination across the cardiac left ventricle was quantified as the counted total number of MBP-ir spots in all nerve bundles at three levels of the left ventricle, the base (top), middle, and apex (bottom), and in four anatomical regions (septal, anterior, lateral, inferior) within each level (Figure 2(a); see Supplementary Figure S1 for details on anatomy). Although MBP-ir spot counts showed high between-subject variability (Supplementary Table S1), a cardioanatomical pattern was present across animals. The level of the left ventricle evaluated had a significant impact on the amount of MBP-ir present (*p* < 0.001). The median number of MBP-ir spots per region in the base (median = 26, IQR = 4.5–49.25) was much higher than in the apex (median = 5, IQR = 1.75–17.75). Pairwise comparisons indicated that MBP-ir spot counts were statistically significantly higher in the base than the apex in the anterior (*p* < 0.014) region and were also close to significantly higher than the apex (*p* < 0.054) and middle (*p* < 0.054) levels in the septal region. Anatomical effects were weaker across regions; while the overall effect of the region was also statistically significant (*p* < 0.001), none of the pairwise differences between individual regions were statistically significant. No differences were found between cardiac levels or regions for the percentage of nerve bundles with any MBP-ir spots (Figure 2(b)).

To assess whether the distribution of MBP-ir spots was associated with other features of cardiac anatomy, we compared the percentage of myelinated fibers per bundle, count of nerve bundles, and size (area) of nerve bundles between the base and apex levels. On average, approximately 14% of nerve fibers were myelinated in each bundle (Figure 2(c)), and there was no difference between cardiac levels. The elevated count of MBP-ir spots in the base relative to the apex was concomitant with larger myocardial wall area (septal *p* < 0.005; inferior *p* < 0.04; Supplementary Figure S1) and increased number of nerve bundles (*p* < 0.001; septal region, *p* < 0.05; inferior region; Figure 2(d) and Supplementary Figure 4(b) in the base of the left ventricle. In contrast, no difference was found between base and apex for size (area) of nerve bundles (Figure 2(e); Supplementary Figure 4(a)). It should be noted that the PGP9.5 bundle size dataset does indicate that the largest bundles 500 *μ*m^2^ were found almost exclusively in the base, not the apex, of the left ventricle (Figure 2(e)). Additionally, a positive correlation was found between bundle size and MBP-ir spot number (Supplementary Figure 4(c)), while a negative correlation existed between bundle size and % of fibers myelinated (Supplementary Figure 4(d)).

### 3.2. Cardiac Sympathetic Neurodegeneration Was Associated with Loss of MBP

**Figure 3:**
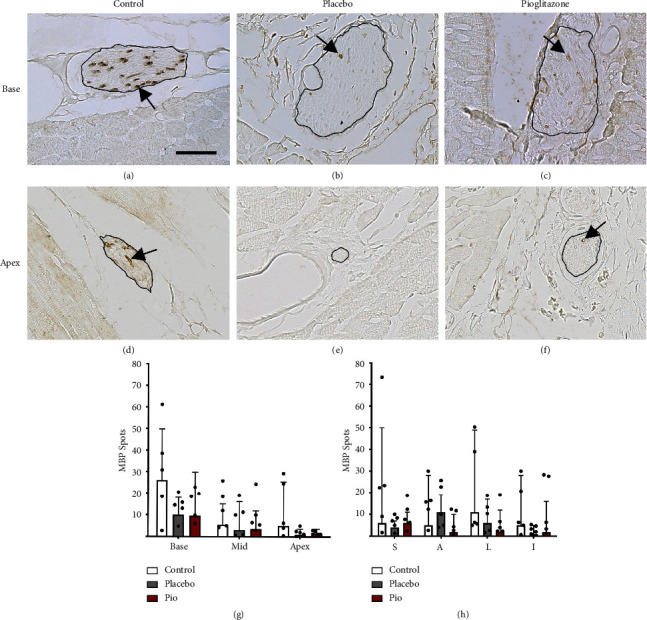
Cardiac MBP-immunoreactive (-ir) spot number is significantly decreased following systemic 6-OHDA. Photomicrographs (a–f) of MBP-ir spots (black arrows) in myocardial nerve bundles (outlined in black) in the cardiac base level (a–c) or apex level (d–f) in normal control (a, d), 6-OHDA + placebo-treated (b, e), or 6-OHDA + pioglitazone-treated (c, f) animals. Scale = 25 *μ*m. Bar graphs (g, h) of the median number of MBP-ir spots counted in each level (g) or region (h) in each treatment group. Error bars show the interquartile range. Black points represent individual animal means; see manuscript results text for statistics. MBP, myelin basic protein; pio, pioglitazone; Mid, middle cardiac level; S, septal; A, anterior; L, lateral; I, inferior.

MBP-ir spots were present, but visibly reduced, in animals treated with 6-OHDA to model PD cardiac sympathetic loss (Figure 3) (for a detailed description of sympathetic denervation in these animals, see [14, 15]). The average number of MBP-ir spots was lower in the placebo- (median = 4, IQR = 1–4) and pioglitazone-treated animals (median = 2, IQR = 1-2) compared to controls (median = 6, IQR = 1–6). Although this main effect of MBP-ir loss was not statistically significant (*p* < 0.068), the effect was significant when considering the interaction of toxin treatment and cardiac level (condition ∗ level *p* < 0.001), as expected if the base and apex were differently impacted. Indeed, the apex of the left ventricle was the cardiac level with the most MBP-ir loss (Figure 3(g)), with 83% fewer total spots counted in placebo-treated and 86% fewer spots in pioglitazone-treated animals relative to controls (control median = 5, IQR = 1.75–17.75; placebo median = 1, IQR = 0–2.5; pioglitazone median = 1.5, IQR = 0–2.5). In the base, 55% fewer total MBP-ir spots were counted in animals in the placebo group and 49% fewer in animals in the pioglitazone group (control median = 26, IQR = 4.5–49.25; placebo median = 10, IQR = 4.75–6; pioglitazone median = 9.5, IQR = 1.75–23.25) compared to normal controls. There were no significant differences between the 6-OHDA + placebo and 6-OHDA + pioglitazone treatment groups.

The cardiac anatomical distribution of the remaining MBP-ir spots in the 6-OHDA-treated monkeys remained similar to the normal control monkeys. In both, the 6-OHDA + placebo and 6-OHDA + pioglitazone groups, the MBP-ir spot distribution was significantly affected by cardiac level (*p* < 0.001) (Figures 3(g) and 3(h)). In the 6-OHDA + placebo animals, the base had significantly more MBP-ir spots than the apex in both the septal (*p* < 0.022) and anterior (*p* < 0.035) regions. Similar to the control group, there was a significant overall effect of region on MBP-ir in the 6-OHDA + placebo animals (*p* < 0.001), but differences between individual regions were not statistically significant. In 6-OHDA + pioglitazone animals, the effect of the cardiac level was statistically significant (*p* < 0.001), while the region was not. MBP-ir spot counts trended toward significantly higher in the base than the apex in the septal (*p* < 0.054) and inferior (*p* < 0.054) regions; counts also trended toward higher in the base than the middle level in the inferior region (*p* < 0.054).

### 3.3. Myelination, Sympathetic Denervation, and PPAR*γ* Activation Showed a Complex Relationship

**Figure 4:**
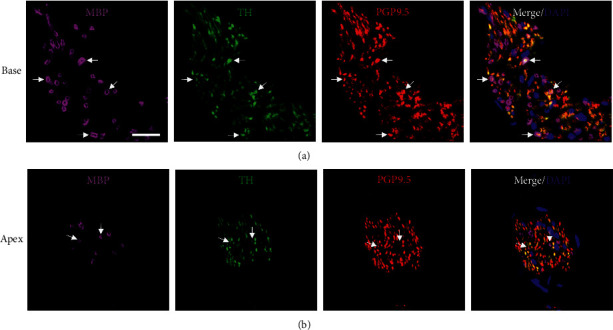
A subset of sympathetic axons in myocardial nerve bundles are myelinated. Photomicrographs of immunofluorescent labeling of MBP, TH, and PGP9.5 in myocardial nerve bundles in the base (a) and apex (b) levels of a control animal. Scale = 50 *μ*m. White arrows point to sympathetic axons (green TH-immunoreactivity (-ir) and red PGP9.5-ir; yellow color) that are myelinated (surrounded by pink MBP-ir). MBP, myelin basic protein; TH, tyrosine hydroxylase; PGP9.5, protein gene product 9.5.

To evaluate the myelination of sympathetic axons in the heart, immunofluorescent labeling of PGP9.5, MBP, and the sympathetic marker tyrosine hydroxylase (TH) was performed in normal, control animals (Figure 4). In myocardial nerve bundles throughout all levels and regions of the left ventricle, a majority of the PGP9.5-ir axons were also immunoreactive for TH, indicating sympathetic fibers. A subset of these TH- and PGP9.5-ir nerve fibers were surrounded by MBP-ir, confirming the presence of myelinated sympathetic nerve fibers in the heart (Figure 4).

Using the values of the normal control group as a baseline, the percent loss of MBP-ir spots, TH-ir in nerve bundles, and TH-ir myocardial fiber density were calculated and compared for each level and region in the 6-OHDA-treated animals. When both toxin-treated groups were combined, the anatomical levels and regions of the left ventricle with greater percent loss of TH-ir myocardial fiber area also tended to have greater percent loss of MBP-ir spots (Spearman *r* = 0.537, *p* < 0.008); this effect trended toward significant for each treatment group separately (placebo Spearman *r* = 0.524, *p* < 0.084; pioglitazone Spearman *r* = 0.552, *p* < 0.067; Supplementary Figures 5(c) and 5(d)). This pattern was not observed when assessing TH-ir inside nerve bundles (Supplementary Figures 5(c) and 5(d)).

**Figure 5:**
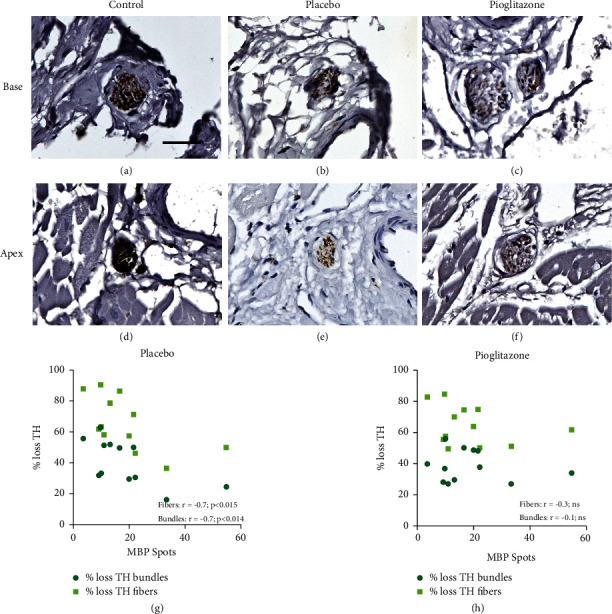
Sympathetic axon loss inversely correlates with the amount of myelin present across cardiac anatomy in placebo- but not pioglitazone-treated animals. Photomicrographs (a–f) of TH-immunoreactivity (-ir) in myocardial nerve bundles in the cardiac base (a–c) or apex (d–f) level in normal control (a, d), 6-OHDA + placebo-treated (b, e), or 6-OHDA + pioglitazone-treated (c, f) animals. Scale = 25 *μ*m. Plots (g, h) of the relationship between MBP-ir spot number and either TH-ir loss in nerve bundles (TH bundles) or loss of TH-ir fibers in the myocardium (TH fibers) in each of the 12 cardiac anatomical areas (3 levels ∗ 4 regions). MBP-ir spot counts are the average (mean) of the control animals. Percent loss of TH-ir in bundles and fibers is the percent difference between the control group average and the 6-OHDA + placebo (g) or 6-OHDA + pioglitazone (h) group average. MBP, myelin basic protein; TH, tyrosine hydroxylase; pio, pioglitazone; ns, not significant.

Finally, to interrogate the role of the presence of myelin in the susceptibility of cardiac nerve fibers and assess for PPAR*γ*-induced neuroprotection, the MBP-ir spot counts in each cardiac level and region of normal control animals were compared to TH-ir percent loss in the 6-OHDA + placebo and 6-OHDA + pioglitazone groups separately. This analysis revealed a statistically significant inverse relationship between MBP-ir spot counts and the severity of sympathetic loss in the 6-OHDA + placebo (TH bundles: Spearman *r* = −0.706, *p* < 0.014; TH fibers: Spearman *r* = −0.699, *p* < 0.015), but not the 6-OHDA + pioglitazone animals (TH bundles: Spearman *r* = −0.315, not significant (ns); TH fibers: Spearman *r* = −0.098; ns) (Figures 5(g) and 5(h)).

## 4. Discussion

The present study demonstrated that myelinated postganglionic sympathetic fibers are heterogeneously present in the left ventricle of rhesus macaques and that systemic 6-OHDA significantly reduced MBP-ir spot counts in the heart. Furthermore, sympathetic neurodegeneration showed an inverse relationship with counts of MBP-ir spots across cardiac anatomy in placebo-, but not pioglitazone-treated, animals.

Based on these findings, three main questions emerge for discussion. First, how does the myelination of axons in the rhesus heart compare to that of humans? Second, are these findings translatable to PD cardiac neurodegeneration? Finally, how does this observed effect of PPAR*γ* activation on cardiac neurodegeneration inform on peripheral neuroprotection?

### 4.1. How Does Myelination of Axons in the Rhesus Heart Compare to That of Humans?

Literature on the myelination status of axons in the heart of any species is sparse [10, 23–26]. To the authors' knowledge, this manuscript is the first such description in rhesus macaques. Studies on myelin in the human heart have been largely limited to the anterior wall of the left ventricle near the left coronary artery [10, 23] and sites proximal to the left ventricle, such as over the roots of coronary arteries near the aortic sinuses and at the coronary groove separating the atria and the ventricles [23]. Furthermore, earlier publications focused their assessment on the epicardial nerve bundles that lie in the adipose tissue on the surface of the myocardium [10, 23]. A review of the literature revealed one publication that performed Sigler's stain to label myelin in whole heart specimens, but this study is difficult to interpret due to the small sample size and what the authors refer to as “dark coloring of the myocardial tissue as simply a by-product of not fully destaining” [25]. In contrast, the present publication assessed myocardial nerve bundles embedded in the myocardial wall of the left ventricle and included samples from multiple levels and regions of the left ventricle. Despite these methodological differences, our findings highlight neuroanatomical similarities between rhesus and human hearts.

Similar to previous descriptions of the distribution of autonomic cardiac innervation in humans [10, 27], nerve fibers in the rhesus left ventricle wall were predominantly sympathetic postganglionic fibers, as indicated by positive immunostaining for the sympathetic marker TH (Figure 5). Although only a subset of TH-ir fibers was myelinated, we identified MBP-ir surrounding multiple TH-ir axons in nearly all nerve bundles in all levels of the heart (Figure 4). Interestingly, autonomic postganglionic fibers in humans are traditionally described as unmyelinated [10, 28, 29]. Additional studies will be needed in humans and nonhuman animal models to determine if myelination of postganglionic sympathetic fibers is different between species or organs. Previous research in rats illustrated that unmyelinated axons in the heart contained significantly more cytoskeletal elements, neurofilaments, and neurotubules, per *μ*m^2^ than samples from the cervical vagal trunk [26]. The authors hypothesized that this could be due to “continuous mechanical stress experienced by these intracardiac nerves” related to the action of the beating heart. It is conceivable that these same unique anatomical and physiological pressures of the heart could also impact myelination, as myelin is known to be neuroprotective [17]. As the focus of this study was sympathetic neurodegeneration and neuroprotection, we limited the assessment of the phenotype of myelinated axons to TH-ir fibers. Parasympathetic postganglionic fibers are also present in the left ventricle, although at only about 15% the frequency of sympathetic fibers [27], and it is unclear how frequently these fibers are myelinated. Primary afferent neurons are also found in the cardiac left ventricle and are typically characterized as including both unmyelinated and myelinated fibers, although unmyelinated axons are significantly more common [30].

Our estimation that approximately 14% of PGP9.5-ir axons in nerve bundles are myelinated is comparable to, although higher than, the 7.8% found in human epicardial nerve bundles [10]. The difference in these values could be due to species and methodological differences, such as the PBS and 4% PFA perfusion performed at the time of nonhuman primate tissue collection. One limitation of this study was the inability to manually count the number of axons in each myocardial nerve bundle when calculating the percentage of myelinated fibers due to insufficient resolution of the imaging method. The percentage of axons myelinated in individual bundles was instead calculated from counted MBP-ir spots and estimated axon number. The axon number estimate was based on a calculated average axon size generated by outlining MBP-ir spots, which were significantly easier to visualize than individual PGP9.5-ir axons. The larger diameter of the myelin sheath than the axon it surrounds could have led to overestimates of the average axon size. For example, previous work has shown that the *g* ratio, the ratio of the inner and outer diameter of the myelin sheath, of myelinated fibers in epicardial bundles ranges widely from approximately 0.2–0.8 [23]. Therefore, the percentage of axons myelinated in myocardial nerve bundles could be lower than the 14% estimate.

### 4.2. Are These Findings Translatable to PD Cardiac Neurodegeneration?

Only one study, to our knowledge, has evaluated the role of myelination in susceptibility of nerve fiber loss in the heart in human disease. The research in PD patients demonstrated that the percentage of unmyelinated axons was 78 ± 9% in the PD group and 92 ± 2% in controls, suggesting that cardiac myelinated axons are somewhat protected in PD [10]. However, it should be noted that the data in the Orimo et al. paper also indicates a considerable loss of myelinated fibers in the hearts of PD patients. Their results state that total axons per mm^2^ decreased from 39300 ± 4650 in controls to 1980 ± 774 in PD. They further demonstrate that this represented a decrease of unmyelinated axons from 34700 ± 2720 in controls to 1620 ± 828 in PD, which then leaves the decrease of myelinated axons to be from approximately 4600 to 360 – a 95% loss of unmyelinated fibers and 92% loss of myelinated fibers in PD. In the present systemic 6-OHDA rhesus model, previously published data illustrated an average 67% loss of cardiac sympathetic fiber density across all levels and regions of the left ventricle [15]. The present work showed that 6-OHDA-induced cardiac sympathetic neurodegeneration was greatest in areas of the heart with the fewest counted MBP-ir spots, suggesting mild myelin-associated neuroprotection against this toxin (Figures 5(g) and 5(h)). Combined with the extensive loss of MBP-ir following 6-OHDA (Figure 3), this finding suggests that the systemic 6-OHDA model of PD cardiac dysautonomia features loss of both myelinated and unmyelinated axons with increased sensitivity of unmyelinated fibers, similar to what was described in PD.

Differences in the degree of myocardial nerve fiber and myelin loss between humans with PD and systemic 6-OHDA-treated monkeys are potentially related to the mechanisms underlying the neurodegeneration. In PD, increased oxidative stress, inflammation, and aggregation of the neuronal protein *α*-synuclein (*α*-syn) forming Lewy bodies are linked to progressive nigral neurodegeneration [31]. In that regard, Lewy bodies have been found in the cardiac plexus [32]. Interestingly, *α*-syn expression in cardiac sympathetic nerves seems to change with PD progression. Abundant *α*-syn is detected in distal axons of sympathetic ganglia of PD patients with intact cardiac sympathetic innervation, but it is decreased with sympathetic neurodegeneration [33]. 6-OHDA is an analog of dopamine that is taken into neurons by catecholamine transporters. After entering the cell, 6-OHDA autoxidizes and interferes with mitochondrial complex I, triggering oxidative stress and inflammation that produce rapid neuronal loss without *α*-syn aggregation [13, 34]. Stereotactic injection of 6-OHDA into the substantia nigra of rats induced magnetic resonance imaging (MRI) detectable neurodegeneration and myelin loss by 1-week time point [35]. Similarly, 6-OHDA-induced cardiac sympathetic loss in the nonhuman primates in this study was detectable by *in vivo* positron emission tomography (PET) at 1 week after toxin [14]. Previously published data from the same nonhuman primates as in the current study showed that 3 months after 6-OHDA, *α*-syn expression in cardiac nerve bundles was not increased [15], which can be interpreted as a difference between the neurotoxin model and PD. Alternatively, it can be argued that, similar to PD patients, *α*-syn expression changes with toxin-induced cardiac sympathetic neurodegeneration, as 6-OHDA may have triggered an increase in *α*-syn that preceded the loss of sympathetic fibers and was resolved by 3 months.

The mechanisms by which myelinated axons are protected relative to unmyelinated are thought to be related to both trophic support from the myelinating Schwann cell and decreased metabolic demand associated with saltatory conduction [17, 36–38]. It should be noted, however, that even unmyelinated axons in the peripheral nervous system are associated with glial cells termed nonmyelinating Schwann cells, or Remak Schwann cells, which provide a barrier between the axon and basal lamina until the fiber reaches its target site [23, 39]. Given the mild neuroprotective role that myelin is proposed to have in both the rhesus heart following 6-OHDA (Figures 5(g) and 5(h)) and in PD [10], future work in other diseases featuring cardiac sympathetic loss such as diabetes [40] or idiopathic REM sleep disorder [41] and pure autonomic failure [42], both proposed to possibly represent prodromal PD [43, 44], is warranted to investigate if similar patterns are observed. This could suggest that therapies aiming to preserve and support myelinating glial cells could have a role in a cardiac neuroprotective strategy across multiple peripheral neuropathies.

### 4.3. How Does This Observed Effect of PPAR*γ* Activation on Cardiac Neurodegeneration Inform on Peripheral Neuroprotection?

Daily oral dosing of the PPAR*γ* agonist pioglitazone following systemic 6-OHDA appeared to prevent the inverse correlation between MBP-ir spot counts and sympathetic fiber loss (Figures 5(g) and 5(h)) across cardiac anatomy. PPAR*γ* is a transcription factor that modulates the expression of genes involved in a variety of cell homeostatic and anti-inflammatory pathways [45, 46]. Previous PET in these animals demonstrated that pioglitazone administration led to mild neuroprotection in the hearts of 6-OHDA-treated animals, particularly at the anterior-lateral region of the base and the septal region of the apex [14]. It should be noted, however, that follow-up histology of sympathetic fibers did not show statistically significant differences between placebo- and pioglitazone-treated monkeys at any individual cardiac region or level [15]. It is currently unclear why pioglitazone administration would disrupt the relationship between cardiac areas having higher myelination and lower neurodegeneration. One potential explanation is that the proneuron survival effects of pioglitazone, while mild in these animals, were more impactful in axons that did not already have the trophic support from myelinating Schwann cells. A related finding has been reported in demyelinated cultured neurons, in which pioglitazone improved axonal mitochondrial transport contributing to neuroprotection [47]. Although we did not observe the preservation of MBP-ir spots in the pioglitazone-treated group, pioglitazone has also been investigated for its role in promoting Schwann cell survival and remyelination [48, 49]. However, these studies either used immortalized cultured Schwann cells [49] or a nerve crush injury model that causes mild axonal degeneration [48]. The considerable and rapid 6-OHDA-induced increase in inflammation, oxidative stress, and loss of the axons needed for myelin to wrap around may have outpaced the protective effects of PPAR*γ* activation on the myelinating Schwann cells in the rhesus hearts.

Research into peripheral neuroprotection in PD is needed, as showcased by the impact that cardiac sympathetic loss has in the daily life of PD patients. Increased blood pressure variability in PD is a consequence of cardiac dysautonomia which leads to a myriad of signs and symptoms, including fatigue and orthostatic hypotension, the latter associated with increased risk of falls and injury [1, 2]. Investigations into preserving peripheral innervation in other diseases point to dietary and lifestyle changes that could also be beneficial to PD patients. For example, studies in diabetic peripheral neuropathy, which is typified by the loss of sensory fibers in the distal leg that progresses proximally, suggest that this neurodegeneration is lessened by administration of vitamin B12 [50, 51]. Exercise has also been shown to reduce diabetic [52–54] and chemotherapy-induced peripheral neuropathy [55–57] in multiple clinical studies. In PD, exercise has been shown to improve motor symptoms associated with nigral cell loss [58–60]. Further exploration of the role of exercise in preserving cardiac innervation is warranted.

## 5. Conclusions

Overall, this study provides a first description of the distribution of myelinated axons in the rhesus cardiac left ventricle and shows similar susceptibility of unmyelinated cardiac axons in PD and the 6-OHDA nonhuman primate model. Future work will be needed to characterize the distribution of myelinated axons in the heart of humans, understand the role of myelin in preserving cardiac innervation in PD, and identify potential pharmacological interventions or lifestyle changes that can preserve cardiac innervation.

## Data Availability

The data used to support the findings of this study are either included within the supplementary information files or are available from the corresponding author upon request.

## Supplementary Materials

Supplementary Materials Supplementary Table 1: MBP-immunoreactive spot count data. Supplementary Figure 1: example images of rhesus macaque cardiac levels and regions and assessment of the average size of each anatomical area. Supplementary Figure 2: intrarater reliability of the MBP-immunoreactive spot counting method. Supplementary Figure 3: photomicrographs of immunolabeling of the pan-neuronal marker PGP9.5 in myocardial nerve bundles in control group animals. Supplementary Figure 4: myocardial nerve bundles in normal, control animals are found in greater numbers at the base than the apex, and the size of bundles shows a complex relationship with myelination. Supplementary Figure 5: there is a trend toward a correlation between loss of TH-immunoreactivity and loss of MBP-ir spots across cardiac anatomy.
