# Supplementary material for: Myelin Basic Protein and Cardiac Sympathetic Neurodegeneration in Nonhuman Primates
Source: Neurol Res Int. 2021 Oct 4;2021:4776610. doi: 10.1155/2021/4776610 (PMC8505074; doi:10.1155/2021/4776610)
Supplement: Supplementary Materials — Supplementary Table 1: MBP-immunoreactive spot count data. Supplementary Figure 1: example images of rhesus macaque cardiac levels and regions and assessment of the average size of each anatomical area. Supplementary Figure 2: intrarater reliability of the MBP-immunoreactive spot counting method. Supplementary Figure 3: photomicrographs of immunolabeling of the pan-neuronal marker PGP9.5 in myocardial nerve bundles in control group animals. Supplementary Figure 4: myocardial nerve bundles in normal, control animals are found in greater numbers at the base than the apex, and the size of bundles shows a complex relationship with myelination. Supplementary Figure 5: there is a trend toward a correlation between loss of TH-immunoreactivity and loss of MBP-ir spots across cardiac anatomy. [file 4776610.f1.pdf]

S Table 1. MBP-immunoreactive spots counted in each region and level of the cardiac left ventricle in each of the 15 subjects in this study. Pio, pioglitazone; mid, middle; MBP, myelin basic protein; 6-OHDA, 6-hydroxydopamine.

	Apex	Mid	Base	Apex	Mid	Base	Apex	Mid	Base	Apex	Mid	Base
	septal			anterior			lateral			inferior		
**Subject**												
**Control 1**	14	6	50	4	28	58	5	10	2	2	4	13
**Control 2**	29	54	137	8	4	25	46	16	55	34	0	28
**Control 3**	5	5	57	3	5	39	51	51	49	39	42	9
**Control 4**	0	0	27	3	6	40	5	12	2	8	2	5
**Control 5**	1	1	3	0	3	5	1	11	3	0	2	0
**6-OHDA + Placebo 1**	1	3	26	1	0	11	6	0	2	0	1	13
**6-OHDA + Placebo 2**	0	6	19	1	33	34	0	26	5	0	11	5
**6-OHDA + Placebo 3**	0	6	15	4	13	13	6	24	26	0	2	4
**6-OHDA + Placebo 4**	5	3	7	0	19	58	15	17	8	0	1	9
**6-OHDA + Placebo 5**	0	0	4	2	2	11	1	1	3	2	0	1
**6-OHDA + Pioglitazone 1**	6	11	39	4	13	20	2	0	18	0	2	2
**6-OHDA + Pioglitazone 2**	1	11	11	2	1	10	2	3	2	0	82	1
**6-OHDA + Pioglitazone 3**	1	2	34	0	1	1	4	3	0	0	6	4
**6-OHDA + Pioglitazone 4**	2	1	4	0	2	33	2	17	38	1	2	16
**6-OHDA + Pioglitazone 5**	6	4	9	4	4	0	0	12	0	0	20	65

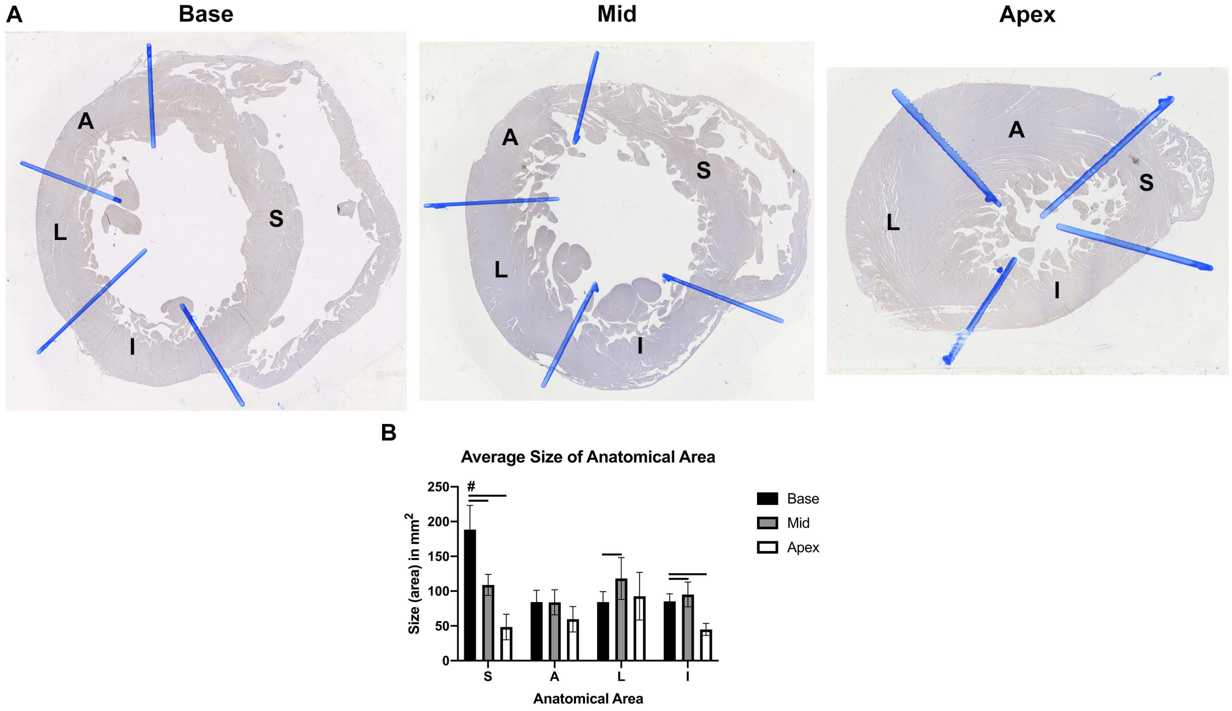

S Figure 1. Example images of rhesus macaque cardiac levels and regions and assessment of the average size of each anatomical area. Scanned images of cardiac tissue (A) immunostained for PGP9.5 and counterstained with hematoxylin on glass slides show representative cardiac levels and regions. After collecting the whole hearts, each heart was sliced into 4mm thick cardiac levels starting from the apex (see details in methods), which represented the cardiac levels. This study focused on three levels: the base level at the top of the left ventricle, the middle level, and the apex level at the bottom of the left ventricle. For analysis of cardiac regions, each tissue slice was subdivided into four regions on the glass slide: septal, anterior, inferior, and lateral. Comparison of the size of the regions in each level (B) showed significant differences between the base and the apex at the septal and inferior regions, a significant difference between the base and the middle at the septal and lateral regions, and a significant difference between the septal region and all other regions at the base level (#) ($p < 0.05$ for all).

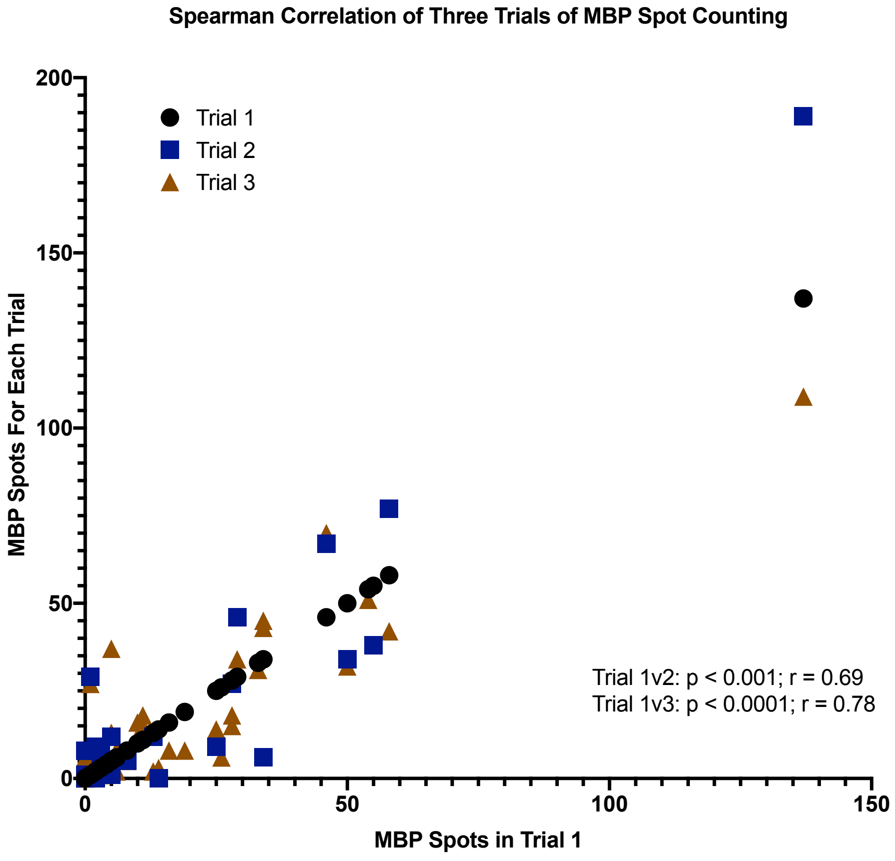

S Figure 2. Intra-rater reliability of the MBP-immunoreactive (-ir) spot counting method. A Spearman correlation was run to assess the degree of correlation between three trials of MBP-ir spot counting in a subset of cardiac tissue. There were statistically significant correlations between the initial trial and both a second and third re-count of MBP-ir spots.

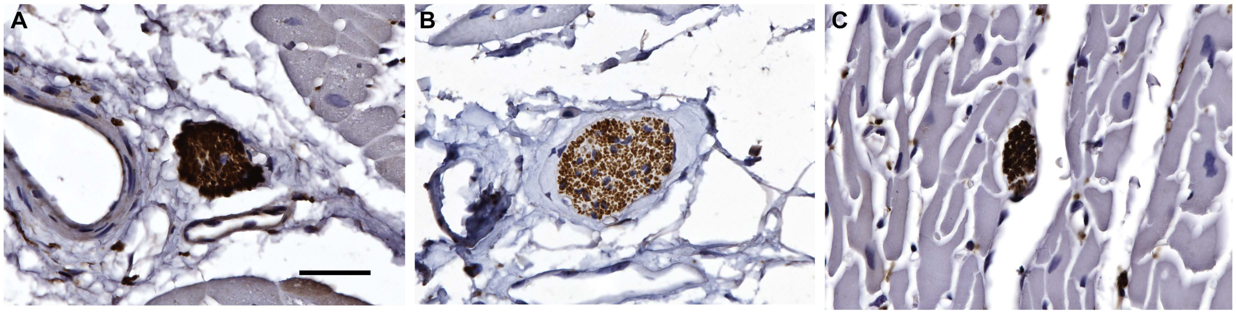

S Figure 3. Photomicrographs of immunolabeling of the pan neuronal marker PGP9.5 (brown) in myocardial nerve bundles in control group animals. Images show examples of bundles at the base (A), middle (B), and apex (C) levels of the left ventricle. Scale bar = 25um. PGP9.5, protein gene product 9.5.

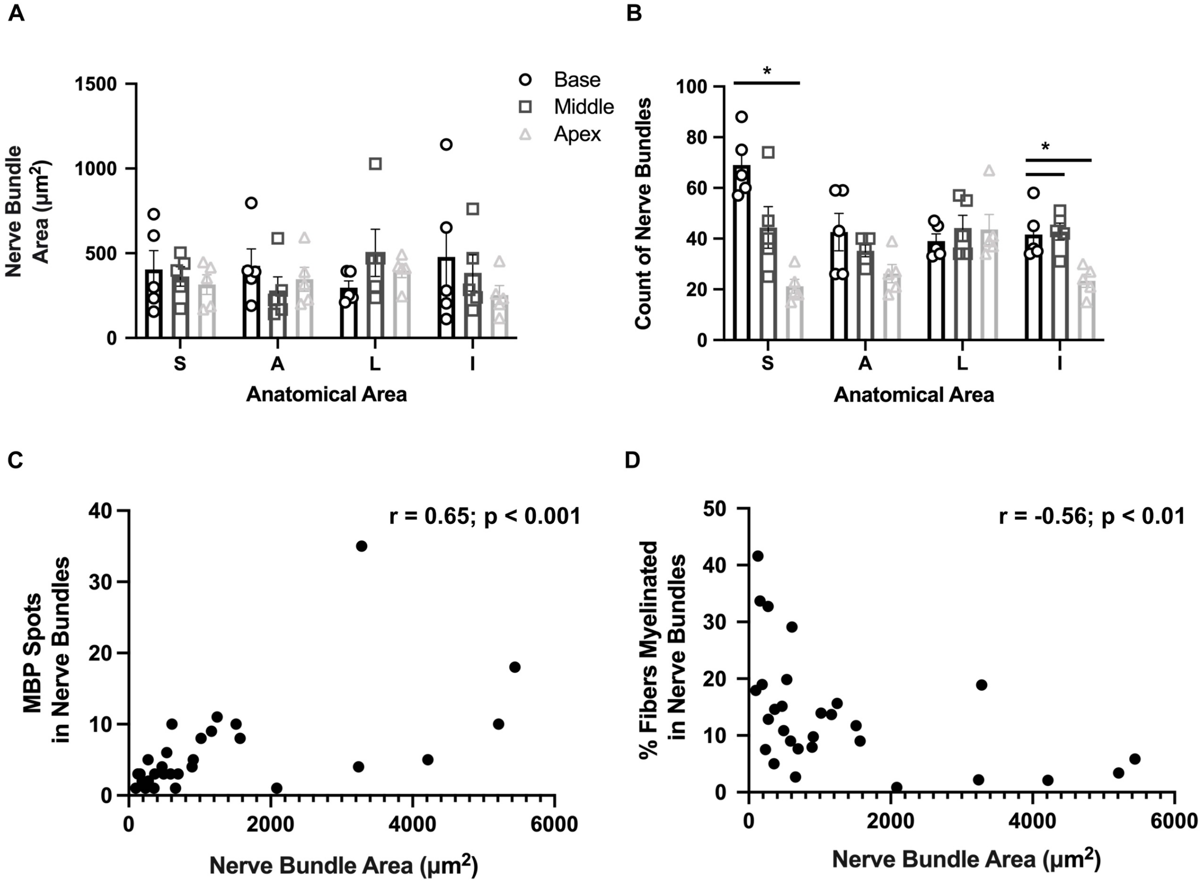

S Figure 4. Myocardial nerve bundles in normal, control animals are found in greater numbers at the base than the apex and size of bundles shows a complex relationship with myelination. Bar graphs of nerve bundle size (A) and number of nerve bundles counted (B) in the twelve anatomical areas (3 levels * 4 regions) assessed. *, a statistically significant difference exists between the two levels indicted by the line in this region. Graphs show mean ± standard error of the mean and each point shows an individual animal. Plots of the relationship between nerve bundle size and the number of MBP-immunoreactive spots in a bundle (C) or the estimated percentage of nerve fibers that are myelinated (D). Note that C and D only include data from the anterior region of 3 control animals. MBP, myelin basic protein; S, septal; A, anterior; L, lateral; I, inferior.

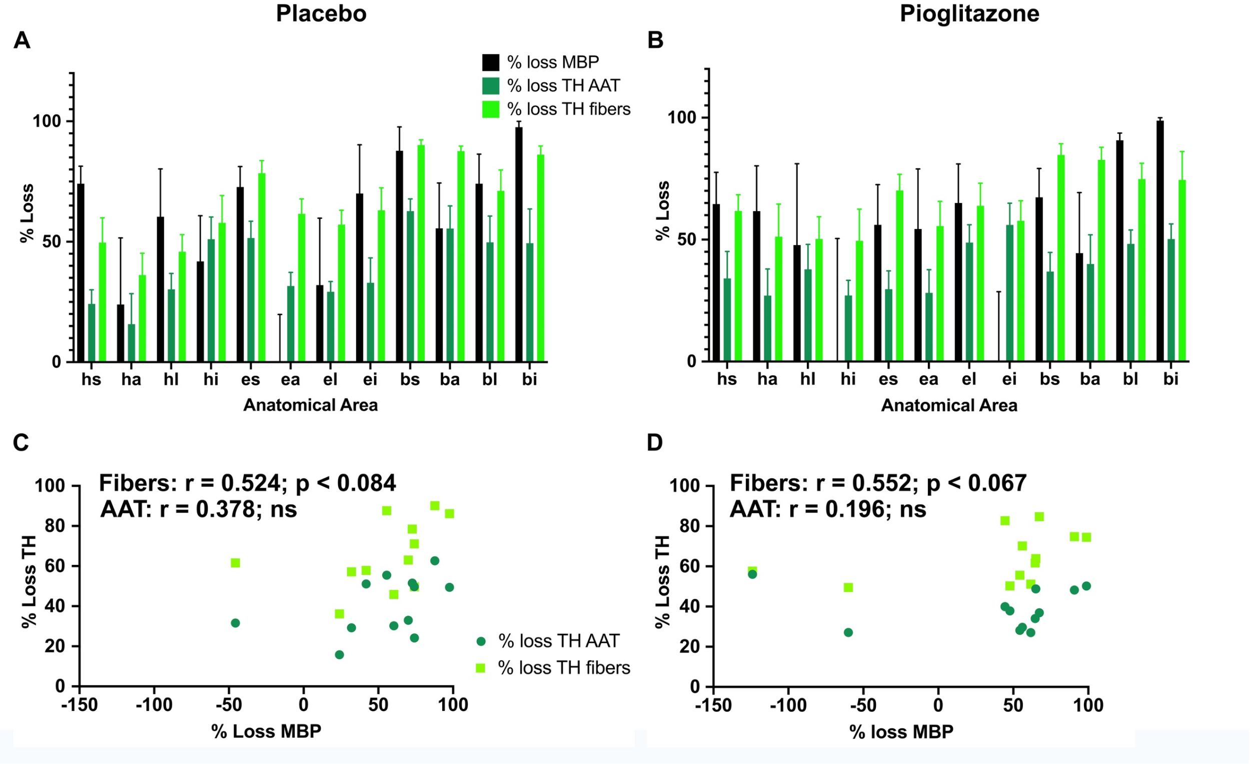

S Figure 5. There is a trend toward a correlation between loss of TH-immunoreactivity (-ir) and loss of MBP-ir spots across cardiac anatomy. Bar graphs (A, B) of the percent loss of MBP-ir spots, TH-ir AAT (TH-ir in nerve bundles), and TH-ir fibers (individual TH-ir nerve fibers in the myocardium) in each of the 12 cardiac anatomical areas (3 levels * 4 regions). Percent loss is the percent difference between the control group average (mean) and the 6-OHDA + placebo (A) or 6-OHDA + pioglitazone (B) group average. Bar graphs show mean ± standard error of the mean. Note that % loss values < 0 are not shown in the graphs. In the X axis labels, 'h' indicates the base level, 'e' middle, and 'b' apex; 's' is the septal region, 'a' anterior, 'l' lateral, and 'I' inferior (e.g., 'hs' is the septal region of the base level, 'bi' is the inferior region of the apex level, etc.). Plots (C, D) of the correlation between loss of MBP and loss of TH in 6-OHDA + placebo- (C) or pioglitazone-treated (D) groups. MBP, myelin basic protein; TH, tyrosine hydroxylase; AAT, % area above threshold; pio, pioglitazone; ns, not significant.
